# Digital Ulcers and Microvascular Abnormalities Presenting As the Initial Manifestations of Pre-scleroderma

**DOI:** 10.7759/cureus.75061

**Published:** 2024-12-03

**Authors:** Alejandro Arango, Reena N Yaman, Sehreen Mumtaz, Andy Abril, Florentina Berianu

**Affiliations:** 1 Department of Internal Medicine, Pontifical Bolivarian University, Medellin, COL; 2 Department of Rheumatology, Mayo Clinic, Jacksonville, USA

**Keywords:** immunosuppressive agents, nailfold videocapillaroscopy, raynaud's phenomenon, scleroderma, vasculopathy

## Abstract

The term Raynaud's phenomenon (RP) is used to describe complex symptoms related to vascular compromise, which are typically exacerbated by cold-induced vasoconstriction, emotional stress, or other sympathomimetic factors. In almost all patients with limited cutaneous systemic sclerosis (SSc), the first symptom is RP, often two to five years before any other symptom of scleroderma. The clinical course and severity of this disease are variable and highly fatal in some individuals, which has led to the development of strategies for timely diagnosis; hence, criteria for the very early diagnosis of systemic sclerosis have been established. Nevertheless, some individuals with this disease may lack classification findings (Raynaud's phenomenon, puffy fingers, and positive antinuclear antibodies), making their condition less apparent. This study reports a different clinical scenario involving a previously healthy 42-year-old woman with recent monophasic RP and digital ulcers in the absence of clinical or immunological manifestations of systemic autoimmune disease. The diagnosis of preclinical systemic sclerosis was made possible based on evidence of capillaroscopic abnormalities. The patient demonstrated favorable evolution following the initiation of treatment with hydroxychloroquine, a statin, and a calcium antagonist. Four years later, she had no recurrences of digital ulcers, a noticeable improvement in her RP, and no clinical features of systemic sclerosis, highlighting the paradigm of the natural history of scleroderma in its preclinical, clinical, advanced severe stages, and immunological abnormalities.

## Introduction

Raynaud's phenomenon refers to a condition characterized by a complex set of symptoms linked to impaired blood flow, usually triggered or worsened by cold temperatures, emotional stress, or substances that mimic the effects of the sympathetic nervous system [1]. It affects approximately 3-5% of the general population, with over 80% of cases representing primary Raynaud's phenomenon (RP) that is not associated with systemic autoimmune disease [2]. Secondary RP is associated with autoimmune disease it is most frequently associated with systemic sclerosis (SSc). Its main complications include digital ulcers, necrosis, and ischemia [2].

Nailfold capillaroscopy is a non-invasive, reproducible, and effective in vivo technique used to visualize and evaluate microcirculation (microscopic vessels) [1,2]. It can be performed in any anatomical location where terminal capillaries are oriented parallel to the skin. Various instruments can be used including dermatoscopes, ophthalmoscopes, or digital videocapillaroscopes that allow precise optical magnification up to 200x [1,2]. This technique is highly useful in studying rheumatic diseases and differentiating between primary and secondary RP, enabling the identification of vascular anomalies such as density, enlargement, presence of giant capillaries, and microhemorrhage which are significant in the disease's natural course [2].

SSc is a systemic autoimmune disease characterized by immune-mediated small vessel vasculopathy and fibrosis of the skin and various organs [3]. Nailfold videocapillaroscopy (NVC) findings pathognomonic for SSc have been described [2,3]. We report the case of a 42-year-old patient with recent-onset RP associated with digital ulceration, without clinical or immunological manifestations suggestive of autoimmune disease, where NVC findings provided the diagnosis of pre-scleroderma.

As this case report involves a single patient and is presented with de-identified data, institutional ethics approval was not required. The patient gave informed consent for the publication of this case, including the use of images and medical history.

## Case presentation

**Figure 1:**
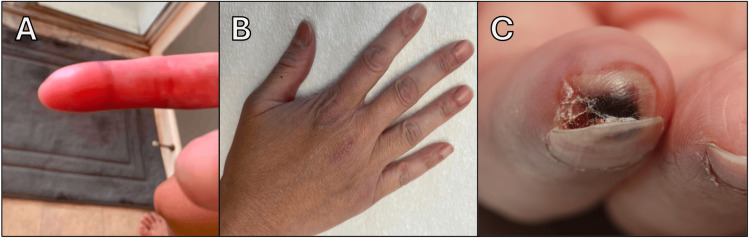
Bilateral Raynaud's phenomenon with vascular involvement. (A) Monophasic Raynaud's phenomenon (redness) on the right index finger. (B) Marked periungual erythema. (C) Digital necrosis and ulceration on the right index fingertip.

A 42-year-old woman with no significant medical history presented to our rheumatology outpatient clinic at Mayo Clinic on May 20, 2020, with a five-month history of bilateral RP affecting her hands (Figure 1A) and feet, with approximately three episodes per day, and periungual erythema (Figure 1B). She also reported noticing a black spot under the nail bed of her right thumb three months prior, associated with local inflammatory signs. However, seven days later, she developed pain, inflammation, and necrosis in the distal phalanx of her right index finger (Figure 1C).

During the rheumatological evaluation, a review of systems revealed no myalgia, asthenia, weight loss, arthralgia, morning stiffness, or symptoms of dryness, and she reported only mild symptoms of gastroesophageal reflux. Physical examination showed normal blood pressure, no alopecia, rash, skin fibrosis, or lymphadenopathy, and no joint swelling or tenderness. Cardiopulmonary and gastrointestinal examinations were unremarkable. The extremities showed no edema or neurological alterations, but active necrosis was present in the right index finger. Laboratory tests revealed mild thrombocytosis, with an otherwise normal complete blood count. Antibody testing was negative for antinuclear antibodies (ANA), antiphospholipid antibodies, and scleroderma-specific antibodies. Other conditions, including systemic lupus erythematosus, Sjögren's syndrome, mixed connective tissue disease, antisynthetase syndrome, cryoglobulinemia, and paraproteinemia secondary to hematological neoplasia, were excluded (Table 1).

**Table 1:** Admission laboratory tests. MCV: mean corpuscular volume; MCH: mean corpuscular hemoglobin; ESR: erythrocyte sedimentation rate; CRP: C-reactive protein; ANA: antinuclear antibodies; SS-A: Sjögren syndrome antigen A; SS-B: Sjögren syndrome antigen B; SM: Smith antibody; RNP: ribonucleoprotein; Scl-70: anti-DNA-topoisomerase I antibody; Jo 1: anti-histidyl tRNA synthetase; C3-C4: complement

Parameters	Patient values	Reference range
Hemoglobin	12.3 g/L	12-15 g/L
Hematocrit	39.7%	35-42%
MCV	80.9 fL	80-100 fL
MCH	25.1 pg per cell	27-33 pg per cell
Platelet count	460,000/mm^3^	150,000-450,000/mm³
ESR	18 mm/h	<20 mm/h
CRP	8 mg/L	<3.0 mg/L
ANA	Negative	Negative
SS-A/Ro	<0.2 U/mL	<25 U/mL
SS-B/La	<0.2	<25 U/mL
Sm	<0.2	<25 U/mL
RNP	<0.2	<20 U/mL
Scl-70	<0.2	<10 U/mL
RNA polymerase III	<0.2 U/mL	<20 U/mL
Jo-1	Negative	Negative
C3	128 mg/dL	88-201 mg/dL
C4	22 mg/dL	15-45 mg/dL
Cryoglobulins	Negative in 72 h	Negative in 72 h
Protein electrophoresis	Normal	N/A

**Figure 2:**
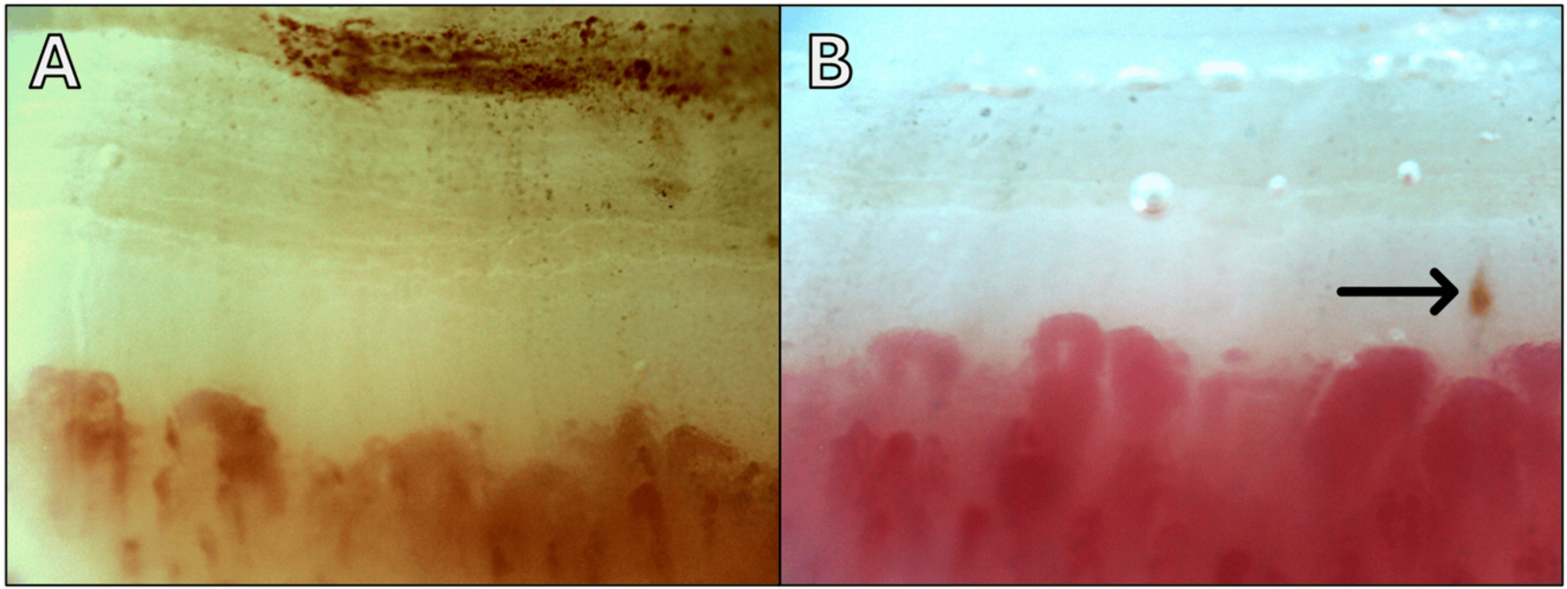
Nailfold videocapillaroscopy showing a capillaroscopy pattern consistent with early scleroderma. (A) Giant capillaries >50 µm in diameter (×200). (B) Capillary microhemorrhages (arrow) and giant capillaries >50 µm in diameter (×200).

NVC was performed for evaluation of her RP (Figures 2A, 2B) revealing a few giant capillaries, microcapillaries, and hemorrhages highly suggestive of systemic sclerosis and consistent with an early scleroderma pattern.

Treatment was initiated with 2% nitroglycerin cream (for three months), hydroxychloroquine 200 mg daily, atorvastatin 40 mg daily, and nicardipine 20 mg every 8 h, continuing to date. Interstitial lung disease and pulmonary hypertension were ruled out through CT chest and transthoracic echocardiogram. The patient showed complete resolution of digital necrosis (over three months) with significant improvement of RP during a four-year follow-up.

## Discussion

In SSc, there is a close pathophysiological and clinical correlation that follows progressive states of endothelial injury and dysfunction [4]. Vascular changes are therefore of high importance when establishing a diagnosis. According to the American College of Rheumatology and the European League Against Rheumatism (ACR/EULAR) in 2013, for classification, at least nine points are required based on the presence of pitting scars, RP, digital ulcers, capillaroscopic abnormalities, telangiectasias, and pulmonary hypertension [5]. However, the clinical spectrum of SSc is quite heterogeneous and may present an unpredictable natural course with some individuals developing mild and stable forms for years, while others develop rapidly progressive or refractory forms with fatal outcomes. Rubio-Rivas et al. report cumulative survival rates of individuals with SSc at 74.9% and 62.5% at five and 10 years, respectively, from the time of diagnosis, highlighting the need for tools to enable early detection and diagnosis [6]. Very early diagnosis of systemic sclerosis (VEDOSS) classification criteria were developed to address this gap [7,8]. However, when analyzing its variables (RP, puffy fingers, positive serology, and capillaroscopic abnormalities), limitations in identifying some individuals with the disease who may lack classification findings, become apparent [9]. This is especially relevant considering that in SSc patients, the presence of RP suggests established vascular dysfunction states that precede the evolution of puffy fingers, sclerodactyly, and the extension of skin involvement [9,10]. Therefore, it is evident that very early clinical manifestations already reflect disease progression that could potentially be irreversible. Hence, identification and treatment in early and potentially subclinical phases of the disease suggest a potentially narrower therapeutic window, intervening before the onset of sclerodactyly, skin fibrosis, or organ damage, which is crucial [11]. Even so, identifying patients in the oligosymptomatic phase that precedes VEDOSS represents a real clinical challenge, although initiating early treatment may be a window of opportunity for prevention of progressive or severe manifestations involvement [11].

In almost all patients with limited cutaneous SSc, the first symptom is RP, often two to five years preceding any other symptom of scleroderma [10]. In this population, the preclinical phase would be defined by the presence of any non-Raynaud's symptoms or include RP itself, but without other manifestations of scleroderma, as was the case with our patient [10]. However, there is still no clear consensus on its precise definition. Nonetheless, in this scenario, accessible and minimally invasive diagnostic aids, such as NVC, are of great help during an early diagnostic approach, as there is an established association between the duration of the disease and microvascular involvement [12]. Shenavandeh et al. in their cohort did not find an association between capillaroscopic patterns and cutaneous subtypes of SSc but did observe a significant association when analyzing disease duration [13]. Interestingly, the early scleroderma pattern was more frequently observed in subjects with limited SSc. However, in the context of VEDOSS, even in preclinical SSc, there is no adequate characterization of capillaroscopic abnormalities, limiting the correct interpretation of the study in this population.

Nonetheless, Cutolo et al. in their preliminary analysis of NVC in VEDOSS, the capi-vedoss experience (n=1085), found a higher frequency of an early scleroderma pattern in this population, as was seen in our patient, suggesting a possible role for NVC in very early stages of the disease [14]. Still, further studies are needed to reach a conclusion supported by evidence [15].

Regarding the immunological profile of these patients, the findings in the literature are extremely varied and controversial. For example, Salazar et al. in their cohort compared subjects with ANA-positive versus ANA-negative SSc (n=3249), finding that the latter presented notably less cutaneous, pulmonary, and especially vasculopathic involvement [16]. This widely differs from the presentation in our patient, who, despite having negative ANA, showed severe microvascular involvement (digital ulceration, giant capillaries, and nailfold bed hemorrhages).

In the present case, the diagnostic approach was challenging, particularly due to the absence of findings suggestive of a specific autoimmune disease as the underlying cause of Raynaud's phenomenon. Among the main differential diagnoses, systemic lupus erythematosus was ruled out due to the absence of multisystemic or cutaneous involvement and the negativity of antinuclear antibodies. Symptoms indicative of mixed connective tissue disease were also absent, and cryoglobulinemia was excluded. Similarly, Sjögren's syndrome was ruled out based on the absence of sicca symptoms as well as cutaneous, neurological, or immunological abnormalities. Neoplastic processes were also excluded due to the lack of symptoms suggestive of malignancy, considering the patient's age, sex, and risk factors. Finally, no paraproteinemia was identified through protein electrophoresis. Although features such as swollen fingers, sclerodactyly, microstomia, telangiectasias, acro-osteolysis, and skin fibrosis were absent, and the specific autoimmune profile was negative for scleroderma, nailfold videocapillaroscopy proved to be highly useful. This technique facilitated the diagnosis of pre-scleroderma by identifying an early scleroderma pattern, providing evidence of microvascular damage in the context of Raynaud's phenomenon secondary to systemic sclerosis. This finding is particularly relevant given the lack of unified criteria for the very early diagnosis of systemic sclerosis (VEDOSS) and the even greater scarcity of information on pre-scleroderma, a concept still under development.

Lastly, the treatment of SSc will depend on the clinical manifestations of each patient, as there is no unified management strategy for very early scleroderma, pre-scleroderma, or isolated microvascular involvement beyond the use of calcium channel blockers or, depending on the severity of vascular obstruction from recurrent and prolonged vasospasm, the use of vasodilators [17]. Other pharmacological groups, such as statins have been considered for their anti-inflammatory effects and reduction of C-reactive protein, low-density lipoprotein concentration, tumor necrosis factor-alpha, and interferon-gamma [18]. Although there is no evidence supporting the use of hydroxychloroquine in the treatment of severe secondary RP, it could play an immunomodulatory role in the vascular lumen [19]. Basta et al. in their study the authors evaluated the response to hydroxychloroquine in subjects with SSc compared to individuals who did not receive the intervention [20]. After three months, they found that participants treated with the antimalarial exhibited a significant reduction in the NEMO score, microhemorrhages, microthrombosis, giant capillary score, and levels of E-selectin, VCAM, and endothelin-1 [20]. These findings support the hypothesis that hydroxychloroquine may be a therapeutic option for preventing microvascular complications in systemic sclerosis [20]. However, further studies with greater statistical power are necessary to establish this intervention as a standard treatment for patients with SSc.

**Figure 3:**
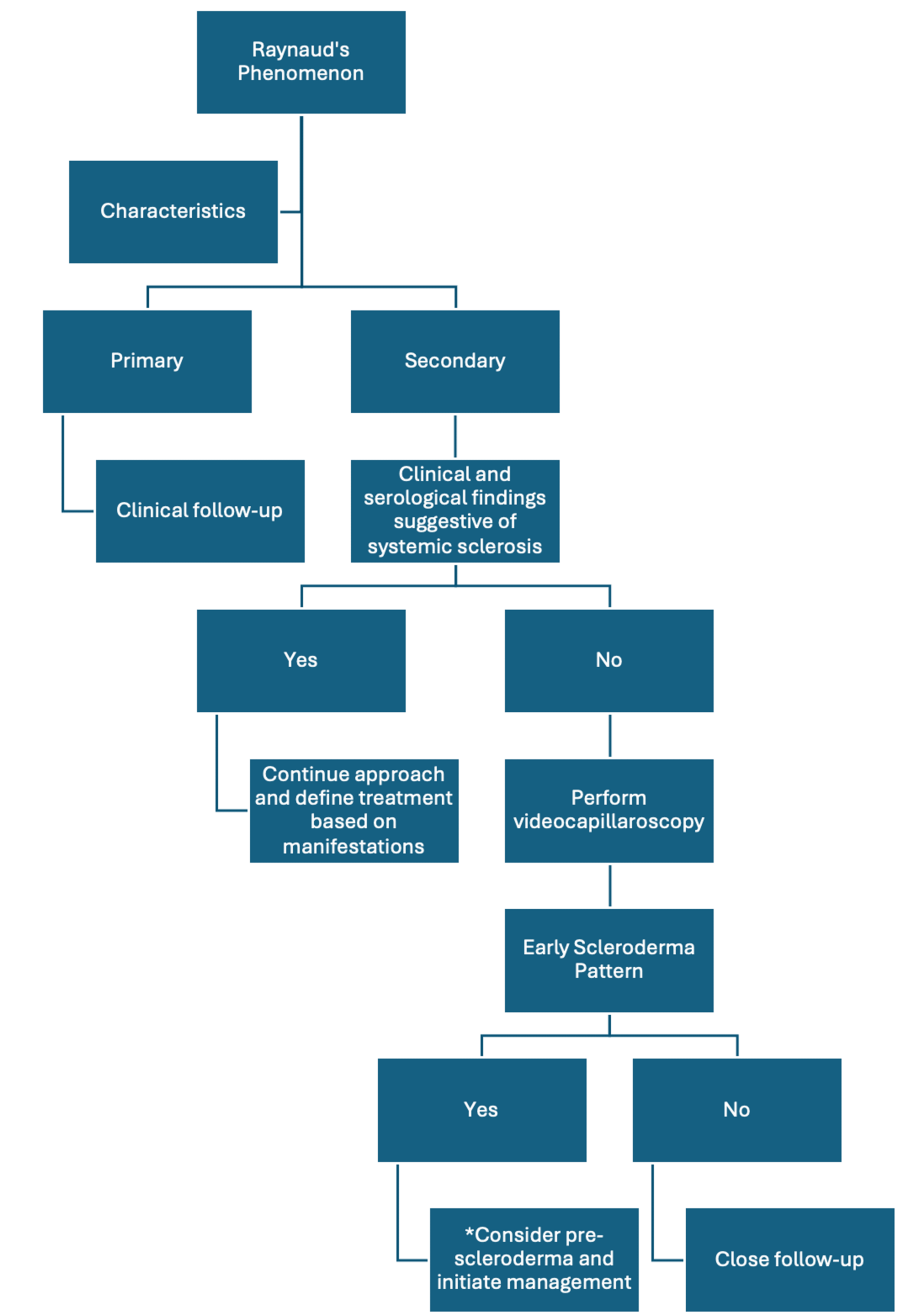
Pre-scleroderma: diagnostic and therapeutic approach. *Treatment includes hydroxychloroquine (200-400 mg daily), high-intensity statin, and calcium channel antagonist. The image is created by the author of this study.

The present case highlights the unusual presentation of our patient, which differs markedly from the typical clinical manifestations of systemic sclerosis and even more so from those of patients with very early systemic sclerosis. This underscores the heterogeneity of the disease and emphasizes the importance of recognizing the concept of "pre-scleroderma," characterized by subtle disease manifestations, even in the absence of immunological alterations. Based on the successful outcome of the present case, we propose the following diagnostic and therapeutic approach, focusing on Raynaud's phenomenon as an epiphenomenon of autoimmunity and microvascular damage (Figure 3).

## Conclusions

In summary, we present a case of a patient with Raynaud's phenomenon and digital ulceration as a sentinel event, accompanied by an NVC pattern highly suggestive of early scleroderma, in the absence of other non-Raynaud's-related manifestations or positivity for specific antibodies. This report underscores the clinical significance of Raynaud's phenomenon as the first manifestation of systemic sclerosis and highlights the crucial role of NVC in the initial diagnostic approach to pre-scleroderma. Moreover, it suggests hydroxychloroquine as a potential treatment strategy for managing and preventing the progression of microvascular damage in this population, in combination with other adjuvant medications.
